# The molecular machinery of meiotic recombination

**DOI:** 10.1042/BST20230712

**Published:** 2024-02-13

**Authors:** Linda Chen, John R. Weir

**Affiliations:** Structural Biochemistry of Meiosis Group, Friedrich Miescher Laboratory, Max-Planck-Ring 9, 72076 Tübingen, Germany

**Keywords:** chromosomes, DNA replication and recombination, meiosis

## Abstract

Meiotic recombination, a cornerstone of eukaryotic diversity and individual genetic identity, is essential for the creation of physical linkages between homologous chromosomes, facilitating their faithful segregation during meiosis I. This process requires that germ cells generate controlled DNA lesions within their own genome that are subsequently repaired in a specialised manner. Repair of these DNA breaks involves the modulation of existing homologous recombination repair pathways to generate crossovers between homologous chromosomes. Decades of genetic and cytological studies have identified a multitude of factors that are involved in meiotic recombination. Recent work has started to provide additional mechanistic insights into how these factors interact with one another, with DNA, and provide the molecular outcomes required for a successful meiosis. Here, we provide a review of the recent developments with a focus on protein structures and protein–protein interactions.

## Introduction

**Figure 1.**
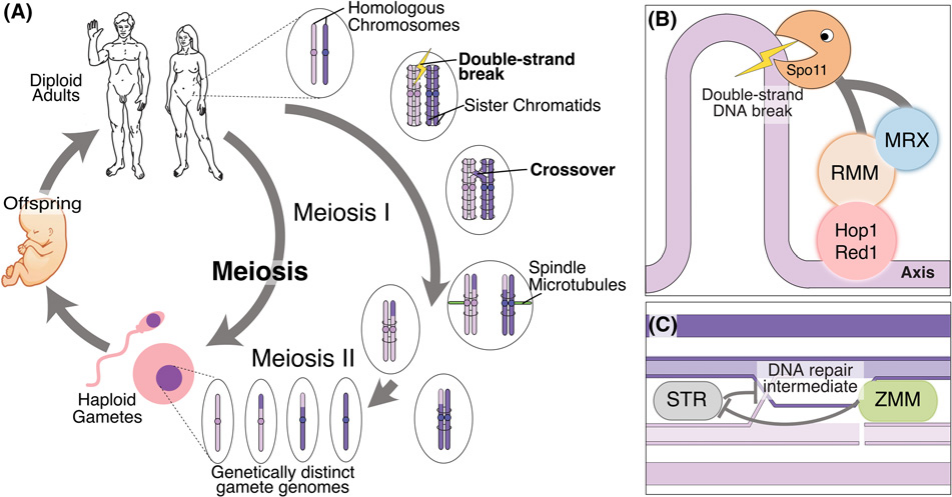
Overview of the key stages in meiosis. (**A**) Cartoon overview of meiosis in the context of the generation and continuation of eukaryotic life. On the right-hand side, cartoon chromosomes are considered to be homologues. The stages of meiosis I are shown as DSB formation, crossover formation and the segregation of homologues. The outcome of meiosis II is shown at the bottom as four genetically distinct haploid gametes. (**B**) Inset of meiotic DSB formation. The axial proteins Hop1 and Red1 recruit the RMM complex proteins. The RMM proteins, together with the MRX, recruit and activate the Spo11 core complex that catalyses meiotic DSB formation in loops of chromatin emerging from the axis. (**C**) The ZMM group of proteins functions to promote meiotic crossover formation by antagonising the activity of anti-crossover factors such as the STR (Sgs1–Top3–Rmi1) complex.

Meiosis, a specialised form of cell division, is key to generating the diversity of life. This process, culminating in the generation of haploid gametes such as eggs, sperm, or spores, facilitates subsequent syngamy, the fusion of these gametes, during fertilisation to create a new euploid organism (Figure 1A). The reduction in genome size during meiosis is achieved through a unique sequence of events: a single round of DNA replication followed by two distinct rounds of chromosomal segregation. Meiosis I segregates homologous chromosomes, while meiosis II, akin to mitosis, segregates sister chromatids.

Physical linkages between chromosomes allow tension to be generated across the bivalent, facilitated by the forces generated by the spindle, and ultimately satisfy the spindle assembly checkpoint [1]. Therefore, such linkages are essential for the faithful segregation of chromosomes. Sister chromatids, segregated during mitosis and meiosis II, are linked by cohesive cohesin that is loaded during DNA replication. During meiosis I, homologous chromosomes do not have intrinsic linkages, and therefore inter-homologue connections must be established prior to the chromosome segregation event during meiosis I, in order that homologous chromosomes be properly sorted and segregated at anaphase I. This will ensure the formation of viable gametes, and subsequent healthy euploid offspring.

Most sexually reproducing species use recombination to link homologous chromosomes, initiated through the programmed formation of double-stranded DNA breaks (DSBs). This mechanism solves the mechanistic conundrum of how to organise homologous chromosomes in meiosis I and simultaneously introduces genetic diversity by reshuffling parental haplotypes. Interestingly, some organisms, like the model nematode *Caenorhabditis elegans*, have decoupled homologous pairing from recombination [2], while others, such as male fruit flies [3], eschew recombination entirely. However, this review will concentrate on meiotic recombination during ‘canonical’ meiosis I, a process common to fungi, plants, and vertebrates.

One defining feature of meiosis I is the formation of a distinct chromosomal architecture: a proteinaceous axis from which loops of chromatin emerge. DSBs, essential for recombination, are introduced in these DNA loops, with the break-forming machinery localised to the axis [4], initiating the process of pairing followed by synapsis. These breaks are repaired using the homologous chromosome rather than the sister chromatid, initiating the process of synapsis — defined by the progressive development of zipper-like connections forming along the chromosome axis. Certain repair intermediates mature into crossovers (COs), the critical junctures where homologous chromosomes exchange arms [5]. Cohesive cohesin complexes between sister chromatids distal to COs sites then provide the necessary physical links between homologues.

**Figure 2.**
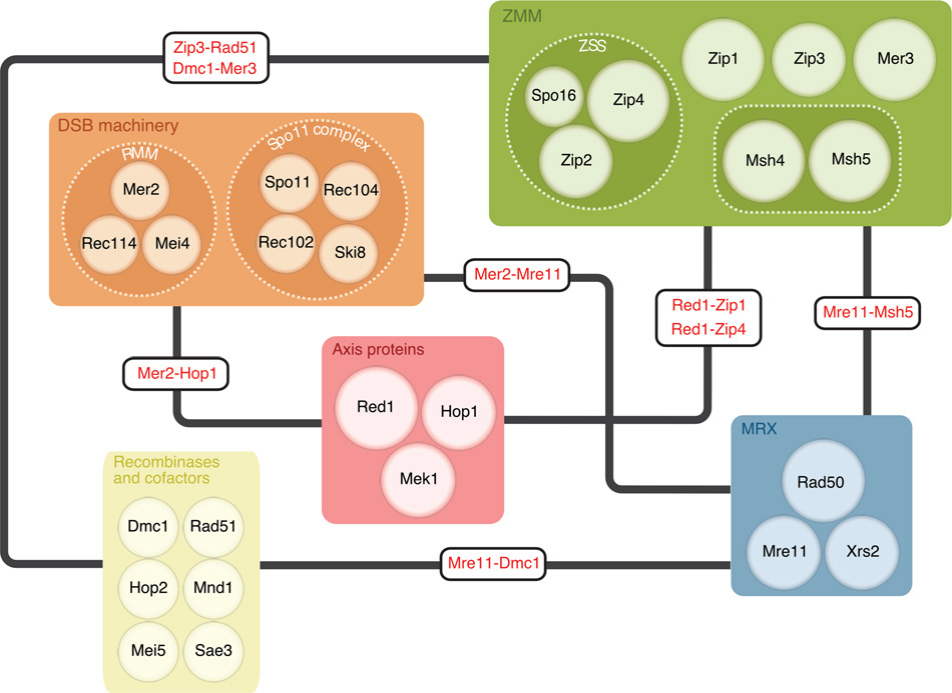
Summary of some key meiotic recombination factors and inter-complex physical interactions in early prophase I. Protein complexes are defined according to the convention described in the main text. Citations for the inter-complex physical interactions are; Zip3–Rad51 [6], Mer2–Mre11 [7], Red1–Zip1 [8], Red1–Zip4 [9] Mer2–Hop1 [7], Mre11–Msh5 [10], Mer3–Dmc1 [11], Mre11–Dmc1 [12].

This minireview aims to concisely delineate our current understanding of the molecular machinery with an emphasis on recent advances and, in particular, protein–protein interactions vital for meiotic recombination (summarised in Figure 2).

## The meiotic axis

At the onset of meiotic prophase, chromosomes undergo a morphological change, as a proteinaceous axis forms along their length, from which chromatin loops emerge [13]. For instance, in *Saccharomyces cerevisiae* (budding yeast), loops are ∼25 kb long [14], while in mice, they range from 1 to 2 Mb [15]. The precise structural organisation of the meiotic axis has not yet been fully elucidated, but it generally comprises at least Rec8-containing cohesin, condensin, a Red1-type axial filament protein (such as Red1 in budding yeast or SYCP2 in mice [16,17]), and one or more HORMA domain proteins such as Hop1 in yeast. The axis is believed to play a pivotal role in determining the proper placement and number of meiotic DSBs, modulating DNA repair, particularly in favouring inter-homologue bias, and in the formation of the synaptonemal complex (SC).

**Figure 3.**
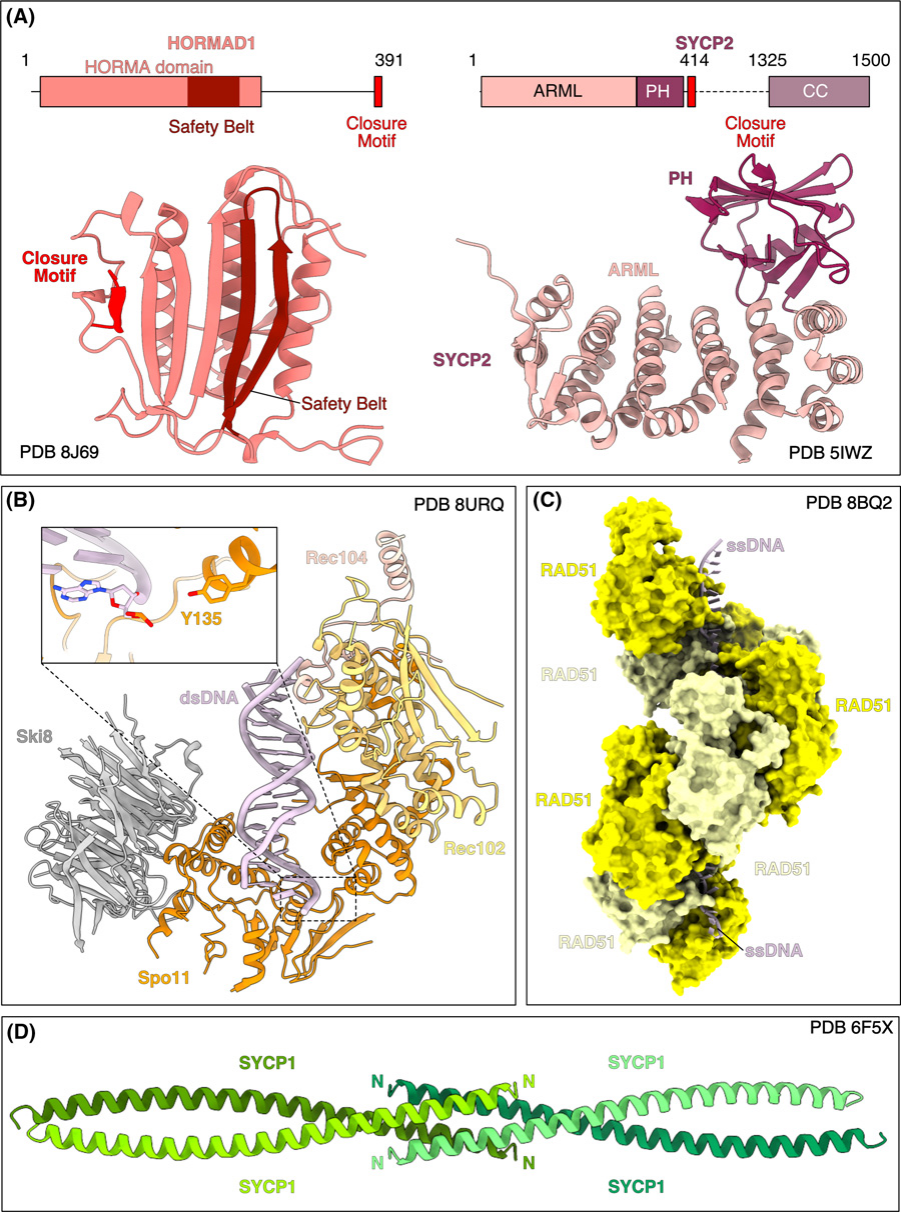
Examples of key experimental protein structures of the meiotic machinery. (**A**) Structures of meiotic axis proteins. Left, X-ray structure of human HORMAD1 (Hop1 in budding yeast). The N-terminal HORMA domain of HORMAD1 physically entraps the C-terminal closure motif (red) due to the movement of the safety belt (maroon) [23]. Right, the crystal structure of mouse SYCP2 N-terminus consisting of the ARM-like (ARML) and PH domains [22], C-terminal to the PH domain is a closure motif [17]. (**B**) CryoEM structure of the *S. cerevisiae* Spo11 core complex (Spo11, orange; Rec102, pale orange; Rec104, pale yellow; Ski8, grey) in complex with dsDNA (pink). The catalytic tyrosine of Spo11 (Y135) is highlighted proximal to the sugar-phosphate backbone of the dsDNA [24]. (**C**) cryoEM structure of human RAD51 bound to single-stranded DNA (pink) [25]. (**D**) Crystal structure of the human SYCP1 (Zip1 in *S. cerevisiae*) αN-end head-to-head assembly region [26].

While the composition of meiotic cohesin varies between species, there seems to invariably exist at least one meiosis-specific kleisin variant — Rec8 [18]. It is thought that Rec8 cohesin contributes to the formation of chromatin loops, through loop extrusion activity [19], but that it also directly recruits Red1-type proteins. Red1 co-IPs with Rec8 [20], and co-localises with cohesin in ChIP-chip [4] and super-resolution microscopy [21], but formal proof of a direct interaction is lacking. Red1/SYCP2 contains an N-terminal globular domain (consisting of an ARM-like and PH domain [22]), of an unknown function (Figure 3A). The C-terminal region of Red1/SYCP2 contains a coiled-coil region with the ability to form both tetramers and higher-order filaments [17]. Red1 is presumed to recruit Hop1-like HORMA domain proteins.

HORMA domains are dynamic domains that can adopt two topologically distinct conformations, open and closed. The transition to the energetically more stable closed state is catalysed by the interaction with a closure motif [27]. Unlike other HORMA domain proteins, the Hop1-like meiotic HORMA domains contain self-binding *cis* closure motifs in their C-terminal region (Figure 3A) [28]. Meiotic HORMAs can also interact with closure motif(s) in *trans* (for example, in Red1-like proteins, Figure 3A, right) but this presumably requires an active remodelling of the HORMA domain through the AAA + ATPase Pch2 (TRIP13 in mammals) [29]. Mammals have two meiotic HORMA domain proteins, HORMAD1 (Figure 3A, left) and HORMAD2 [30], and HORMAD2 has been shown to bind to the SYCP2 closure motif [17].

Hop1-like proteins in many species (though notably not in mammals) contain an additional chromatin binding domain (CBR) consisting of at least a winged-helix-turn helix domain, and in some cases combined with a PHD domain [31]. In yeast, this region can specifically bind to nucleosomes, which provides a second recruitment pathway for Hop1 and Red1 [31]. In budding yeast, one function of the CBR appears to be to enhance DSB formation on small chromosomes through localisation of Hop1 to nucleosome-rich islands [32]. Inversely, the removal of Hop1 is an important mechanism for the suppression of DSB formation in, for example, rDNA repeat regions [33,34]. Thus regulating levels of chromosomal Hop1 locally appears to be a fundamental mechanism for regulating DSB formation.

## Meiotic DNA break formation and initial repair

DSBs, essential to initiate meiotic recombination, are catalysed by the topoisomerase-like enzyme Spo11. The complexity of DSB formation is underscored by its dependence on numerous additional factors; at least nine proteins (in addition to Spo11) in budding yeast are needed for DSB formation [35]. This regulatory framework likely reflects a balance between preventing genome instability due to uncontrolled DSB formation and ensuring sufficient breaks for reliable homologue linkage. Spo11 is similar to the TopoVI family of type II DNA topoisomerases, which require an ‘A’ subunit (here, Spo11) and a ‘B’ subunit for full functionality [36]. DeMassy and co-workers discovered the Spo11 ‘B’ subunit, TOPOVIBL, in mice [37], while at the same time the Grelon laboratory reported the discovery of the plant Spo11 ‘B’ subunit — MTOPVIB — which was found to bind to both plant Spo11 proteins [38]. These discoveries allowed the realisation that a ‘B’ subunit in budding yeast is encoded in the Rec102 protein [37].

Recent work made use of recombinant yeast Spo11 ‘core complex’ (Spo11, Rec102, Rec104, and Ski8), molecular modelling and mass spectrometry to confirm the role of Rec102 as a ‘B’ subunit that functions together with Rec104 [39]. Importantly, this work also showed that the Spo11 complex contains only one copy of the Spo11 subunit; two catalytically active Spo11 subunits would be required to break the backbone of double-stranded DNA. This is in line with the idea that a key role for the additional Spo11-associated factors is to accommodate the dimeriz (or multimerization) of Spo11 to activate it [40,41]. A recent breakthrough from the Keeney laboratory has taken the work with recombinant Spo11 complex a step further and revealed the cryoEM structure of the Spo11 core complex in complex with dsDNA (Figure 3B) [24]. Ski8 is canonically involved in regulating the RNA exosome ‘moonlights’ as part of the yeast Spo11 core complex, where it interacts with Spo11 through the same motif that is also found in Ski3 [42,43], but the role of Ski8 in the Spo11 complex is thought to be restricted to yeasts.

What types of DNA sequence are cleaved by Spo11 complexes? Several extrinsic factors guide the Spo11 machinery to DNA break ‘hotspots’. This includes the concentration of axial proteins (see above), the chromatin state [44] and post-translational modifications on nucleosomes (reviewed in [45]). Recombinant Spo11 core complexes have a preference for binding to bent DNA [39]. Consistent with this, *in vivo* it was also observed that Spo11 has a preference for sequences that match a DNA bending site. Moreover, the periodicity of the break sites observed is consistent with Spo11 cutting on the same face of underwound DNA. Finally, DSB sites correlated with TopoII binding sites, strongly indicating a role for topological stress in DSB site preference [46]. Additional factors play a further role in modulating DSB site selection. The PHD domain protein Spp1, canonically part of the COMPASS methyltransferase complex [47], also targets the meiotic DSB forming machinery to promoter regions through an interaction with H3K4me3 nucleosomes and the Spo11 accessory factor Mer2 [48,49]. In vertebrates, the protein PRDM9 recognises certain DNA sequences via a C-terminal Zn-finger array and also targets the DSB machinery to these loci [50–52].

The association of the Spo11 core complex with the meiotic axis is a key aspect of its functionality (Figure 1C). This interaction is thought to be facilitated by Mer2, a protein capable of binding directly to Hop1 within the chromosome axis [7]. The mammalian ortholog of Mer2, IHO1, also binds directly to the axial protein HORMAD1 [53], and this is facilitated by DDK phosphorylation of the C-terminus of IHO1 [54], consistent with the previously described role of DKK phosphorylation of Mer2 [55–58]. Initially, Mer2 was identified as a component of a complex alongside Rec114 and Mei4, termed the RMM complex [59]. Rec114 and Mei4, including their mammalian counterparts REC114 and MEI4, form a stoichiometric ‘RM’ complex characterised by two Rec114 molecules bound to Mei4 [60]. In mice, the factor ANKRD31 was shown to be a direct interactor of REC114, necessary for normal DSB patterning and essential for recombination in the X/Y pseudoautosomal region (PAR) [61,62].

The interaction between Mer2 and the RM complex adds a layer of complexity to this system. Experiments have shown that in yeast, both Mer2 and the Rec114–Mei4 complex can independently form nucleoprotein condensates on DNA in the presence of a crowding agent [60]. Interestingly, mutations impairing Mer2's ability to bind DNA result in the loss of *in vivo* foci formation and a subsequent decrease in Spo11-induced DSBs [60]. Recent studies have demonstrated that in mice, IHO1 can bind directly to the REC114–MEI4 complex, even in the absence of condensate formation [60,63]. This was also shown for yeast, but the assembly showed low affinity [60]. These discrepancies could come from the differing need for specific post-translational modifications in different species, or might indicate that the stoichiometric mouse RMM complex represents an intermediate stage in the formation of higher-order nucleoprotein condensates.

How is the Spo11 core complex recruited to the RMM complex? Rec102 and Rec104 are known to bind with Rec114, as established through yeast two-hybrid (Y2H) assays [43]. The importance of this interaction is underlined by the observation that mutations in Rec114's N-terminal PH domain disrupt its association with Rec102 and Rec104, as seen in Y2H assays. This disruption is associated with a decrease in Spo11-initiated DSB formation [60]. Further elucidating these interactions, De Massy, Robert, Kadlec, and colleagues have recently demonstrated a direct physical connection between the C-terminus of TOPOVIBL and the N-terminal PH domain of REC114 in mice. Disrupting this interaction results in a loss of DSB formation in female mice and a delayed formation in males [64].

Interestingly both ANKRD31 [61,62] and IHO1 [63] bind to the PH domain of REC114 in a mutually exclusive manner. This presents an apparent paradox for the function of the REC114 PH domain in mice. A more complex assembly might occur through a series of compatible or cooperative interactions. Considering that IHO1 and Mer2 both exist as tetramers and the REC114–MEI4 complex shows a 2:1 stoichiometry, a single IHO1 tetramer might ostensibly recruit four REC114–MEI4 complexes. This arrangement would leave the four PH domain binding sites available for ANKRD31 and TOPOVIBL.

MRX complex (Mre11, Rad50, Xrs2; Figure 3C) is required for the first steps in the DNA damage response and for telomere maintenance in mitotically dividing cells, but it is also required for the creation of meiotic DSBs in budding yeast [65,66] and in *C. elegans* [67], but not in plants [68] or fission yeast [69]. The physical connection between MRX and the Spo11 complex appears to also be mediated by Mer2. Mer2 was shown to interact with Xrs2 in a Y2H experiment [43], and Mer2 was recently shown to interact with Mre11 in a manner dependent on several conserved N-terminal residues in Mer2 [7]. Similarly, in *Arabidopsis*, Mer2 (PRD3) also interacts with Mre11 [70].

Regarding the regulation of the above-described interactions and their precise roles in Spo11 activation, our understanding, particularly of the latter, is still developing. It is anticipated that future structural studies, biochemical assays, and genetic analyses will provide deeper insights. We do have more information about the regulation of these interactions. For instance, Mer2 requires phosphorylation by both Cdk and DDK kinases at its N-terminal region to recruit Rec114 and Mei4, which is essential for DSB formation [4,56,71]. This phosphorylation might enhance Mer2's binding to the Rec114 PH domain. Additionally, the recent discovery that a Mer2 residue crucial for Mre11 interaction undergoes significant SUMOylation in meiosis [72] suggests that SUMOylation might be a key regulator of Mer2's interaction with the MRX complex.

## DNA repair and inter-homologue bias

There are many excellent reviews on the detailed mechanisms of homologous recombination [73–76]. Briefly, once DSB resection has been initiated by Mre11 and Sae2 (CtIP), long-range resection occurs via Exo1 (EXO1), generating long tracts of ssDNA. This ssDNA is initially coated in RPA, before it is exchanged for one of two recombinases, Rad51, which is active in both the soma and the germline, or Dmc1, which is a germline-specific recombinase. ssDNA coated with recombinases are known as presynaptic filaments (Figure 3C), and these are competent to invade dsDNA (generating a displaced ssDNA) to interrogate these regions for sequence homology. In budding yeast, the meiosis-specific factors Mei5–Sae3 promote the exchange of RPA for Dmc [77]. Dmc1 seems to localise preferentially to the cut ends of resected DNA, and Rad51 to the opposite end [78]). Hop2–Mnd1 promotes the strand exchange activity of Dmc1/Rad51 [79,80].

In the mammalian germline, in addition to RPA, ssDNA is also coated with complexes of MEIOB/SPATA22, a heterodimer that forms an arrangement of DNA binding domains analogous to the heterotrimeric RPA complex [81–83]. Exchange of RPA or MEIOB/SPATA22 for RAD51/DMC1 requires BRCA2 [84]. A meiosis-specific binding partner of BRCA2, MEILB2 [85,86] is, together with BRME1 (also known as MEIOK21) [87–91], required for the proper loading of DMC1/RAD51. Intriguingly BRCA2 does not require MEILB2–BRME1 for loading RAD51 in the soma, suggesting that it might facilitate the BRCA2 loading of DMC1 or be required for the removal of MEIOB/SPATA22 in the germline. Indeed the latter might be more likely, since MEILB2–BRME1 binds directly to MEIOB/SPATA22 [86,89].

The replicated sister chromatid is an ideal template to repair DSBs, with repair from the sister in somatic cells being some 4-fold more frequent than from the homologue [92]. However, inter*sister* recombination events are non-productive for the formation of inter-homologue COs and during meiosis repair from the homologue is ∼5-fold more frequent than from the sister [93,94]. This inversion of DNA repair frequency is known as inter-homologue bias.

The axial proteins play an important role in the establishment of inter-homologue bias. Removal of Red1 reverts the meiotic bias and gives rise to a mitotic-like DNA repair [95]. The S/T kinase Mek1 is recruited to the axis in response to DSB formation, by binding to the ATM/ATR phosphorylation site on Hop1 [96–99]. Mek1 phosphorylates both Rad54 and Hed1, which attenuate Rad51 activity [100,101]. How might this enable Mek1 to contribute to inter-homologue bias? One model proposes that Mek1 kinase activity is spatially restricted to the axis, and DNA repair is suppressed in a zone of influence around the initial break site. This zone of DNA repair suppression includes the proximal (and aligned) sister chromatid, but the homologous chromosome is presumably outside this Mek1 sphere of influence [102]. One issue with this model is that Mek1 also phosphorylates global targets, especially the transcription factor Ndt80, which prevents binding to target sequences and thus prevents progression through meiosis until DNA damage has been resolved [103,104].

## ZMM proteins in crossover formation

The formation of germline COs is crucial in most organisms to establish the specific physical linkages between homologous chromosomes necessary for satisfying the spindle assembly checkpoint. However, COs are generally detrimental in somatic cells and are thus typically disfavoured [105]). Nascent DNA repair intermediates are often disassembled by the STR (Sgs1–Top3–Rm1) complex (Figure 1C). A group of meiosis-specific proteins, collectively known as ZMM, play a pivotal role in stabilising DNA repair intermediates and channelling them towards pathways more likely to result in COs (refer to Table 1 for details). These ZMM proteins were identified due to the shared impact of mutations on CO formation and synapsis [121,122]. We will briefly explore what is currently known about the different ZMM factors.

**Table 1.** Core recombination proteins

Protein group	Protein name in			Structural description	Functional description
	*S. cerevisiae*	*M. musculus*	*A. thaliana*		
Axial proteins	Red1	SYCP2 (and SYCP3)	ASY3	N-terminal ARM domain followed by a PH domain [16,22]. Closure motif for interaction with Hop1 [106]. C-terminal coiled-coil region [17]	Speculated to interact with cohesin, may also interact with centromere proteins [22]. Forms a filament that likely is the basis of the axis and subsequent axial element of the SC [17]
	Hop1	HORMAD1 and HORMAD2	ASY2	N-terminal HORMA domain [107] with C-terminal closure motif [106]. Additional chromatin binding domain in some organisms	Involved in DSB formation. Involved in the activation of the meiotic checkpoint
	Mek1	?	?	N-terminal FHA domain, C-terminal S/T kinase domain	Meiotic checkpoint effector. Inhibits Rad51 via Rad54 and Hed1 phosphorylation. [101] Prevents progression through meiosis via Ndt80 phosphorylation [103]
RMM complex	Mer2	IHO1	PRD3	Coiled-coil forming a parallel homotetramer [108]	Binding to several factors including Spp1 [48,49], Rec114 and Mei4, Hop1, Mre11, and nucleosomes [7]
	Rec114	REC114	PHS1	N-terminal PH domain [61,109], C-terminal homodimerization region [60,108,110]	Phosphorylation of Rec114 down-regulates DSB formation [111]
	Mei4	MEI4	PRD2	N-terminus of Mei4 forms a globular structure, C-terminus consists of HEAT repeats [108]	N-term of Mei4 binds to two Rec114 DNA binding domains [63,108–110]
Spo11 complex	Spo11	SPO11	SPO11-1 and SPO11-2	Topoisomerase-like (Top6A) factor	Catalyses the formation of meiotic DSBs by cleavage of the phosphodiester backbone [112,113]
	Rec102 and Rec104	TOPOVIBL	MTOPIVB	Rec102 similar to the transducer domain of the B subunit, and Rec104 replaces the GHKL domain [24,39]	Presumably activates and regulates Spo11 activity. Likely forms further protein–protein interactions.
ZMM	Mer3	HFM1	MER3	Helicase core with additional domains, similar to Brr2 helicase [11]	Stabilises early DNA intermediates by D-loop extension [114]
	Zip11	SYCP1	ZYP1a and ZYP1b	Coiled-coil protein; tetramer that self-assembles at N- and C-terminal ends into a lattice [26]	Transverse filament component of SC. N-terminus associated with central element of SC, C-terminus with the meiotic axis [115]
	Zip2	SHOC1/ZIP2	SHOC1	C-terminal is structurally similar to XPF [9,116]	Binding to structured DNAs
	Zip3	HEI10 and RNF212/RNF212B	HEI10	N-terminal RING domain, C-terminal coiled-coil	SUMO or ubiquitin ligase. Plant HEI10 is the master regulator of crossover number and distribution [117]
	Zip4 (Spo22)	TEX11	ZIP4	TPR repeat protein	Likely interaction hub for the ZMM proteins and additional recombination components [9]
	Spo16	SPO16	PTD	Structurally similar to ERCC1 [9,116]	Exact function unknown, likely structured DNA binding [9]
	Msh4	MSH4	MSH4	Structurally similar to bacterial MutS, i.e. a ring structure with a large central channel [118]	Binds to DNA repair intermediates and stabilises them [119,120]
	Msh5	MSH5	MSH5		

The proteins discussed in this minireview are described here and, where known or present, the orthologous proteins from *S. cerevisiae*, *Mus musculus*, and *Arabidopsis thaliana* are shown.

1 As outlined in the text, Zip1 is functionally a ‘ZMM’ but also the major component of the transverse filament of the synaptonemal complex.

Zip2, Zip4, and Spo16 together form a complex known as ZZS [9]. Within this complex, Zip2 and Spo16 interact to form a heterodimer [116], structurally akin to the XPF–ERCC1 nuclease (Figure 3E), albeit lacking endonuclease activity [9,116]. *In vitro* studies reveal that the Zip2–Spo16 complex has an affinity for DNA, particularly structured or bent DNA forms. Zip4, characterised by its TPR repeat structure, binds to the N-terminal region of Zip2 [9]. TPR repeat proteins are often structural scaffolds that interact with a wide range of peptide motifs [123]. Consistent with this function, it was found that Zip4 not only interacts with the axial protein Red1 in Y2H assays but Red1 also shows strong enrichment in Zip4 IP-MS experiments [9]. Furthering our understanding, recent work has shown that Zip4 also directly binds to central element proteins of the SC, specifically through Ecm11 [124]. This study from the Borde laboratory is particularly significant as it for the first time elucidates the physical connection between ZMM proteins and the central element of the SC.

Mer3 is a helicase with many extra domains beyond its helicase core. It is most closely related to the spliceosomal RNA helicase Brr2 [11,125]. The helicase activity of Mer3 has been previously suggested to expand nascent D-loops, thus stabilising them. Indeed, *in vitro* Mer3 clearly has a strong preference for D-loop DNA [11,126]. The preference for D-loop DNA binding suggests that Mer3 may bind to early recombination intermediates. This is supported by *in vivo* data that shows Mer3 loci forming early in meiotic prophase [127] and with a higher number of foci than subsequent COs forming [128].

*In vivo* mutations that abrogate the helicase activity of Mer3 result in mild CO phenotypes, in contrast with the deletion of Mer3 [126,129]. In the spliceosome, the extra domains of Brr2 contribute to protein–protein interactions, and it seems likely to be similar for Mer3; the Ig-like domain of Mer3 contributes to the direct binding of Mlh1–Mlh2 (MutLβ) [126]. The Mer3–MutLβ complex functions to constrain D-loop extension through binding to, and inhibiting Pif1 helicase, thus reducing the size of gene conversion tracts [126,130]. We recently discovered that Mer3 can also bind to the meiotic recombinase Dmc1, and to the Top3–Rmi1 complex which is involved in the disassembly of DNA repair intermediates [11]. It is currently unclear whether Mer3 has further direct physical connections to the ZMM proteins, or if this is mediated through DNA substrates.

Msh4 and Msh5, collectively known as MutSγ, form a heterodimer that is structurally and functionally akin to the bacterial DNA mismatch repair factor MutS, which is characterised by its ring-like structure [118]. *In vitro,* studies demonstrate MutSγ’s preference for binding to double Holliday junctions (dHJs), though it generally exhibits high affinity for a variety of DNA repair intermediates [119,120]. This binding is believed to physically entrap two duplexes of double-stranded DNA, thereby stabilising the recombination intermediate. However, super-resolution microscopy data suggest that the Msh4/5 complex may only embrace one dsDNA in the recombination intermediate [131].

After they have been established, dHJs need to be resolved prior to the removal of cohesive cohesin from chromosomal arms at anaphase I. The resolution of dHJs can result in either non-crossover (NCO) or CO formation. In most model organisms the majority of meiotic COs are generated through the activity of the MutLγ endonuclease, a complex of Mlh1 and Mlh3, the activity of which exclusively generates COs [132]. MutLγ is not a structure-specific endonuclease [133], though it does preferentially bind Holliday Junctions [134]. How then does MutLγ only generate COs? Two recent studies from the Hunter and Cejka laboratories revealed that MutLγ endonuclease activity is stimulated *in vitro* by EXO1, PCNA, and RFC [135,136]. These findings lead to a model which proposes that the asymmetry of PCNA retained at joint molecules might provide a signal that stimulates MutLγ endonuclease to generate COs.

Significant gaps in our understanding of the function of ZMM proteins remain, leaving fundamental questions unanswered. Key among these are the mechanisms by which specific DSB sites are ‘selected’ by ZMM proteins, the exact order of binding events among these proteins, and the intricate details of how the temporal and spatial organisation of the ZMM interactome is controlled, particularly in relation to post-translational modifications.

## Synapsis and crossover distribution

In the study of meiosis across a broad range of organisms, a common observation is the simultaneous occurrence of CO formation and the physical ‘zippering’ or synapsis of homologous chromosomes. This process is mediated by the SC, a structure integral to this pairing. Synapsis typically begins at DSB sites and progresses along the chromosomal axis [137]. The COs, as discussed earlier, occur within the SC, which connects to and forms the axial element of the SC. The intricate structure, function, and implications of the SC in disease have been comprehensively reviewed recently [115]. The SC is composed of three gross morphological elements, the central element, which runs along the midline of the SC, the axial element, which is a remodelled meiotic axis in the context of the SC, and the transverse filaments which link the axial and central elements. Numerous recent structural studies from the Davies laboratory have provided insight into the detailed organisation of the SC. Highlights include the revelation that part of the central element can polymerise by itself, forming intermediate filament-like structures [138], and details of the tetramerization regions of SYCP1 that form the gross structural arrangement of the transverse filaments [26] (Figure 3F).

The distribution of COs, a topic of current interest in meiotic research, was thoroughly reviewed in this journal [139]. However, a brief summary is pertinent. In most organisms, CO distribution is not random. The occurrence of a CO at one locus typically reduces the probability of another CO nearby, a phenomenon termed ‘crossover interference’. The ZMM proteins are primarily responsible for generating these interfering, or class I, COs. Among other factors, a group of key regulators in this process are RING E3 ligases belonging to two related families, Zip3 and HEI10. In *S. cerevisiae* only e Zip3 is present [6], whereas plants and *Sordaria macrospora* only have the HEI10 member [140]. Mammals have both HEI10 and the Zip3-related RNF212 [141,142] (and the paralog RNF212B) (Table 1). On synapsed chromosomes, HEI10 forms loci that exhibit ‘coarsening,’ where their size increases as their number decreases [117,143,144]. The mechanisms and regulatory processes behind HEI10 coarsening are currently under active investigation, promising to unveil further insights into the complex orchestration of CO distribution, and perhaps offering the possibility of exogenously manipulating CO numbers.

## Conclusions and outlook

Meiotic recombination is an essential process, required at the organismal level to facilitate the proper segregation of homologous chromosomes, and at the species level to continually generate new allele combinations. The fundamental mechanism of meiosis — the breaking and subsequent modified repair of the genome — is a high-risk strategy. To ensure the necessary outcome, without compromising genome integrity, requires the temporal and spatial coordination of a variety of meiosis-specific factors that modify and act in consort with somatic factors.

One of the challenges of studying any cellular process is pleiotropic mutant effects, especially with the use of deletions. Thus one goal must be the generation of separation of function mutants. The recent protein structure prediction revolution, spearheaded by AlphaFold2 [145] has considerably lowered the barriers to high-resolution structural information necessary for point mutant design. From this, separation-of-function mutants can be used to study the details of meiotic recombination, as has been demonstrated recently [63,108,110]. At the time of writing, advanced prediction algorithms like AlphaFold2 do not yet possess the capability to model post-translational modifications, small molecule ligands, or nucleic acids. However, given the rapid advancements in this field, it's plausible to anticipate that these features will be integrated shortly. Far from rendering *in vitro* biophysical and biochemical studies obsolete, the advent of AlphaFold2 underscores their importance. These studies are crucial not only for validating the predictions of such algorithms but also for providing detailed input for complex components and stoichiometries, which are essential for accurate modelling.

## Perspectives

- Meiotic recombination is at the very centre of the continuation and diversity of eukaryotic life.
- It will be necessary to explore the relationships between different subcomplexes of the meiotic machinery and understand the contributions made by various post-translational modifications.
- Large-scale biochemical reconstitutions will explore the role of each meiotic factor in a reductionist approach, while CryoET will provide detailed images of meiotic machines *in situ*.

## Open Access

Open access for this article was enabled by the participation of Max Planck Digital Library in an all-inclusive *Read & Publish* agreement with Portland Press and the Biochemical Society under a transformative agreement with MPDL.

## Abbreviations

CBR: chromatin binding domain
dHJs: double Holliday junctions
DSBs: double-stranded DNA breaks
SC: synaptonemal complex
Y2H: yeast two-hybrid

## References

[1] McAinsh, A.D. and Kops, G.J.P.L. (2023) Principles and dynamics of spindle assembly checkpoint signalling. Nat. Rev. Mol. Cell Biol. 24, 543–559 10.1038/s41580-023-00593-z 36964313

[2] Rog, O. and Dernburg, A.F. (2013) Chromosome pairing and synapsis during *Caenorhabditis elegans* meiosis. Curr. Opin. Cell Biol. 25, 349–356. 10.1016/j.ceb.2013.03.003 23578368 PMC3694717

[3] McKee, B.D., Yan, R. and Tsai, J.H. (2012) Meiosis in male Drosophila. Spermatogenesis 2, 167–184 10.4161/spmg.21800 23087836 PMC3469440

[4] Panizza, S., Mendoza, M.A., Berlinger, M., Huang, L., Nicolas, A., Shirahige, K. et al. (2011) Spo11-accessory proteins link double-strand break sites to the chromosome axis in early meiotic recombination. Cell 146, 372–383 10.1016/j.cell.2011.07.003 21816273

[5] Zickler, D. and Kleckner, N. (2023) Meiosis: dances between homologs. Annu. Rev. Genet. 57, 1–63 10.1146/annurev-genet-061323-044915 37788458

[6] Agarwal, S. and Roeder, G.S. (2000) Zip3 provides a link between recombination enzymes and synaptonemal complex proteins. Cell 102, 245–255 10.1016/s0092-8674(00)00029-5 10943844

[7] Rousova, D., Nivsarkar, V., Altmannova, V., Raina, V.B., Funk, S.K., Liedtke, D. et al. (2021) Novel mechanistic insights into the role of Mer2 as the keystone of meiotic DNA break formation. Elife 10, e72330 10.7554/eLife.72330 34951404 PMC8848140

[8] Cheng, C.H., Lo, Y.H., Liang, S.S., Ti, S.C., Lin, F.M., Yeh, C.H. et al. (2006) SUMO modifications control assembly of synaptonemal complex and polycomplex in meiosis of *Saccharomyces cerevisiae*. Genes Dev. 20, 2067–2081 10.1101/gad.1430406 16847351 PMC1536058

[9] De Muyt, A., Pyatnitskaya, A., Andréani, J., Ranjha, L., Ramus, C., Laureau, R. et al. (2018) A meiotic XPF-ERCC1-like complex recognizes joint molecule recombination intermediates to promote crossover formation. Genes Dev. 32, 283–296 10.1101/gad.308510.117 29440262 PMC5859969

[10] Uetz, P., Giot, L., Cagney, G., Mansfield, T.A., Judson, R.S., Knight, J.R. et al. (2000) A comprehensive analysis of protein-protein interactions in *Saccharomyces cerevisiae*. Nature 403, 623–627 10.1038/35001009 10688190

[11] Altmannova, V., Firlej, M., Müller, F., Janning, P., Rauleder, R., Rousova, D. et al. (2023) Biochemical characterisation of Mer3 helicase interactions and the protection of meiotic recombination intermediates. Nucleic Acids Res. 51, 4363–4384. 10.1093/nar/gkad175 36942481 PMC10201424

[12] Krogan, N.J., Cagney, G., Yu, H., Zhong, G., Guo, X., Ignatchenko, A. et al. (2006) Global landscape of protein complexes in the yeast *Saccharomyces cerevisiae*. Nature 440, 637–643 10.1038/nature04670 16554755

[13] Møens, P.B. and Pearlman, R.E. (1988) Chromatin organization at meiosis. Bioessays 9, 151–153 10.1002/bies.950090503 3071365

[14] Schalbetter, S.A., Fudenberg, G., Baxter, J., Pollard, K.S. and Neale, M.J. (2019) Principles of meiotic chromosome assembly revealed in *S. cerevisiae*. Nat. Commun. 10, 4795 10.1038/s41467-019-12629-0 31641121 PMC6805904

[15] Patel, L., Kang, R., Rosenberg, S.C., Qiu, Y., Raviram, R., Chee, S. et al. (2019) Dynamic reorganization of the genome shapes the recombination landscape in meiotic prophase. Nat. Struct. Mol. Biol. 26, 164–174. 10.1038/s41594-019-0187-0 30778236 PMC6403010

[16] Tromer, E.C., Wemyss, T.A., Ludzia, P., Waller, R.F. and Akiyoshi, B. (2021) Repurposing of synaptonemal complex proteins for kinetochores in Kinetoplastida. Open Biol. 11, 210049 10.1098/rsob.210049 34006126 PMC8131943

[17] West, A.M.V., Rosenberg, S.C., Ur, S.N., Lehmer, M.K., Ye, Q., Hagemann, G. et al. (2019) A conserved filamentous assembly underlies the structure of the meiotic chromosome axis. Elife 8, e40372 10.7554/elife.40372 30657449 PMC6349405

[18] Klein, F., Mahr, P., Galova, M., Buonomo, S.B.C., Michaelis, C., Nairz, K. et al. (1999) A central role for cohesins in sister chromatid cohesion, formation of axial elements, and recombination during yeast meiosis. Cell 98, 91–103 10.1016/S0092-8674(00)80609-1 10412984

[19] Kim, E., Barth, R. and Dekker, C. (2023) Looping the genome with SMC complexes. Annu. Rev. Biochem. 92, 15–41 10.1146/annurev-biochem-032620-110506 37137166

[20] Sun, X., Huang, L., Markowitz, T.E., Blitzblau, H.G., Chen, D., Klein, F. et al. (2015) Transcription dynamically patterns the meiotic chromosome-axis interface. Elife 4, e07424 10.7554/eLife.07424 26258962 PMC4530585

[21] Köhler, S., Wojcik, M., Xu, K. and Dernburg, A.F. (2017) Superresolution microscopy reveals the three-dimensional organization of meiotic chromosome axes in intact *Caenorhabditis elegans* tissue. Proc. Natl Acad. Sci. U.S.A. 114, E4734–E4743 10.1073/pnas.1702312114 28559338 PMC5474826

[22] Feng, J., Fu, S., Cao, X., Wu, H., Lu, J., Zeng, M. et al. (2017) Synaptonemal complex protein 2 (SYCP2) mediates the association of the centromere with the synaptonemal complex. Protein Cell 8, 538–543 10.1007/s13238-016-0354-6 28150150 PMC5498334

[23] Wang, H., Xie, R., Niu, F., Yang, Q., An, L., Wu, C. et al. (2023) Structural and biochemical insights into the interaction mechanism underlying HORMAD1 and its partner proteins. Structure 31, 1578–1588.e3 10.1016/j.str.2023.09.006 37794593

[24] Yu, Y., Wang, J., Liu, K., Zheng, Z., Arter, M., Bouuaert, C.C. et al. (2023) Cryo-EM structure of the Spo11 core complex bound to DNA. bioRxiv 2023.10.31.564985 10.1101/2023.10.31.564985v1 PMC11746154 39304764

[25] Appleby, R., Bollschweiler, D., Chirgadze, D.Y., Joudeh, L. and Pellegrini, L. (2023) A metal ion-dependent mechanism of RAD51 nucleoprotein filament disassembly. iScience 26, 106689. 10.1016/j.isci.2023.106689 37216117 PMC10192527

[26] Dunce, J.M., Dunne, O.M., Ratcliff, M., Millán, C., Madgwick, S., Usón, I. et al. (2018) Structural basis of meiotic chromosome synapsis through SYCP1 self-assembly. Nat. Struct. Mol. Biol. 25, 557–569 10.1038/s41594-018-0078-9 29915389 PMC6606445

[27] Gu, Y., Desai, A. and Corbett, K.D. (2022) Evolutionary dynamics and molecular mechanisms of HORMA domain protein signaling. Annu. Rev. Biochem. 91, 541–569 10.1146/annurev-biochem-090920-103246 35041460

[28] Vader, G. and Musacchio, A. (2014) HORMA domains at the heart of meiotic chromosome dynamics. Dev. Cell 31, 389–391 10.1016/j.devcel.2014.11.009 25458007

[29] Vader, G. (2015) Pch2(TRIP13): controlling cell division through regulation of HORMA domains. Chromosoma 124, 333–339 10.1007/s00412-015-0516-y 25895724

[30] Wojtasz, L., Daniel, K., Roig, I., Bolcun-Filas, E., Xu, H., Boonsanay, V. et al. (2009) Mouse HORMAD1 and HORMAD2, two conserved meiotic chromosomal proteins, are depleted from synapsed chromosome axes with the help of TRIP13 AAA-ATPase. PLoS Genet. 5, e1000702 10.1371/journal.pgen.1000702 19851446 PMC2758600

[31] Milano, C.R., Ur, S.N., Gu, Y., Tromer, E.C., Zhang, J., Hochwagen, A. et al. (2023) Chromatin binding by HORMAD proteins regulates meiotic recombination initiation. bioRxiv 2023.03.04.531117. https://www.biorxiv.org/content/10.1101/2023.03.04.531117v1 10.1038/s44318-024-00034-3 PMC10907721 38332377

[32] Heldrich, J., Milano, C.R., Markowitz, T.E., Ur, S.N., Vale-Silva, L.A., Corbett, K.D. et al. (2022) Two pathways drive meiotic chromosome axis assembly in *Saccharomyces cerevisiae*. Nucleic Acids Res. 50, 4545–4556 10.1093/nar/gkac227 35412621 PMC9071447

[33] Vader, G., Blitzblau, H.G., Tame, M.A., Falk, J.E., Curtin, L. and Hochwagen, A. (2011) Protection of repetitive DNA borders from self-induced meiotic instability. Nature 477, 115–119 10.1038/nature10331 21822291 PMC3166416

[34] San-Segundo, P.A. and Roeder, G.S. (1999) Pch2 links chromatin silencing to meiotic checkpoint control. Cell 97, 313–324 10.1016/s0092-8674(00)80741-2 10319812

[35] Yadav, V.K. and Claeys Bouuaert, C. (2021) Mechanism and control of meiotic DNA double-strand break formation in *S. cerevisiae*. Front. Cell Dev. Biol. 9, 642737 10.3389/fcell.2021.642737 33748134 PMC7968521

[36] Buhler, C., Gadelle, D., Forterre, P., Wang, J.C. and Bergerat, A. (1998) Reconstitution of DNA topoisomerase VI of the thermophilic archaeon *Sulfolobus shibatae* from subunits separately overexpressed in *Escherichia coli*. Nucleic Acids Res. 26, 5157–5162 10.1093/nar/26.22.5157 9801313 PMC147979

[37] Robert, T., Nore, A., Brun, C., Maffre, C., Crimi, B., Bourbon, H.M. et al. (2016) The TopoVIB-Like protein family is required for meiotic DNA double-strand break formation. Science 351, 943–949 10.1126/science.aad5309 26917764

[38] Vrielynck, N., Chambon, A., Vezon, D., Pereira, L., Chelysheva, L., Muyt, A.D. et al. (2016) A DNA topoisomerase VI–like complex initiates meiotic recombination. Science 351, 939–943 10.1126/science.aad5196 26917763

[39] Bouuaert C, C., Tischfield, S.E., Pu, S., Mimitou, E.P., Arias-Palomo, E., Berger, J.M. et al. (2021) Structural and functional characterization of the Spo11 core complex. Nat. Struct. Mol. Biol. 28, 92–102 10.1038/s41594-020-00534-w 33398171 PMC7855791

[40] Diaz, R.L., Alcid, A.D., Berger, J.M. and Keeney, S. (2002) Identification of residues in yeast Spo11p critical for meiotic DNA double-strand break formation. Mol. Cell Biol. 22, 1106–1115 10.1128/MCB.22.4.1106-1115.2002 11809802 PMC134631

[41] Sasanuma, H., Murakami, H., Fukuda, T., Shibata, T., Nicolas, A. and Ohta, K. (2007) Meiotic association between Spo11 regulated by Rec102, Rec104 and Rec114. Nucleic Acids Res. 35, 1119–1133 10.1093/nar/gkl1162 17264124 PMC1851646

[42] Halbach, F., Reichelt, P., Rode, M. and Conti, E. (2013) The yeast ski complex: crystal structure and RNA channeling to the exosome complex. Cell 154, 814–826 10.1016/j.cell.2013.07.017 23953113

[43] Arora, C., Kee, K., Maleki, S. and Keeney, S. (2004) Antiviral protein Ski8 is a direct partner of Spo11 in meiotic DNA break formation, independent of its cytoplasmic role in RNA metabolism. Mol. Cell 13, 549–559 10.1016/s1097-2765(04)00063-2 14992724

[44] Pan, J., Sasaki, M., Kniewel, R., Murakami, H., Blitzblau, H.G., Tischfield, S.E. et al. (2011) A hierarchical combination of factors shapes the genome-wide topography of yeast meiotic recombination initiation. Cell 144, 719–731 10.1016/j.cell.2011.02.009 21376234 PMC3063416

[45] Borde, V. and de Massy, B. (2013) Programmed induction of DNA double strand breaks during meiosis: setting up communication between DNA and the chromosome structure. Curr. Opin. Genet. Dev. 23, 147–155 10.1016/j.gde.2012.12.002 23313097

[46] Prieler, S., Chen, D., Huang, L., Mayrhofer, E., Zsótér, S., Vesely, M. et al. (2021) Spo11 generates gaps through concerted cuts at sites of topological stress. Nature 594, 577–582 10.1038/s41586-021-03632-x 34108684

[47] Shilatifard, A. (2012) The COMPASS family of histone H3K4 methylases: mechanisms of regulation in development and disease pathogenesis. Annu. Rev. Biochem. 81, 65–95 10.1146/annurev-biochem-051710-134100 22663077 PMC4010150

[48] Acquaviva, L., Székvölgyi, L., Dichtl, B., Dichtl, B.S., André, C..L., Nicolas, A. et al. (2013) The COMPASS subunit Spp1 links histone methylation to initiation of meiotic recombination. Science 339, 215–218 10.1126/science.1225739 23160953

[49] Sommermeyer, V., Béneut, C., Chaplais, E., Serrentino, M.E. and Borde, V. (2013) Spp1, a member of the Set1 complex, promotes meiotic DSB formation in promoters by tethering histone H3K4 methylation sites to chromosome axes. Mol. Cell 49, 43–54 10.1016/j.molcel.2012.11.008 23246437

[50] Baudat, F., Buard, J., Grey, C., Fledel-Alon, A., Ober, C., Przeworski, M. et al. (2010) PRDM9 is a major determinant of meiotic recombination hotspots in humans and mice. Science 327, 836–840 10.1126/science.1183439 20044539 PMC4295902

[51] Parvanov, E.D., Petkov, P.M. and Paigen, K. (2010) Prdm9 controls activation of mammalian recombination hotspots. Science 327, 835 10.1126/science.1181495 20044538 PMC2821451

[52] Myers, S., Bowden, R., Tumian, A., Bontrop, R.E., Freeman, C., MacFie, T.S. et al. (2010) Drive against hotspot motifs in primates implicates the PRDM9 gene in meiotic recombination. Science 327, 876–879 10.1126/science.1182363 20044541 PMC3828505

[53] Stanzione, M., Baumann, M., Papanikos, F., Dereli, I., Lange, J., Ramlal, A. et al. (2016) Meiotic DNA break formation requires the unsynapsed chromosome axis-binding protein IHO1 (CCDC36) in mice. Nat. Cell Biol. 18, 1208–1220 10.1038/ncb3417 27723721 PMC5089853

[54] Dereli, I., Telychko, V., Papanikos, F., Raveendran, K., Xu, J., Boekhout, M. et al. (2023) Seeding the meiotic DNA break machinery and initiating recombination on chromosome axes. bioRxiv 10.1101/2023.11.27.568863 PMC10997794 38580643

[55] Murakami, H. and Keeney, S. (2014) Temporospatial coordination of meiotic DNA replication and recombination via DDK recruitment to replisomes. Cell 158, 861–873 10.1016/j.cell.2014.06.028 25126790 PMC4141489

[56] Sasanuma, H., Hirota, K., Fukuda, T., Kakusho, N., Kugou, K., Kawasaki, Y. et al. (2008) Cdc7-dependent phosphorylation of Mer2 facilitates initiation of yeast meiotic recombination. Genes Dev. 22, 398–410 10.1101/gad.1626608 18245451 PMC2216698

[57] Wan, L., Niu, H., Futcher, B., Zhang, C., Shokat, K.M., Boulton, S.J. et al. (2008) Cdc28-Clb5 (CDK-S) and Cdc7-Dbf4 (DDK) collaborate to initiate meiotic recombination in yeast. Genes Dev. 22, 386–397 10.1101/gad.1626408 18245450 PMC2216697

[58] Matos, J., Lipp, J.J., Bogdanova, A., Guillot, S., Okaz, E., Junqueira, M. et al. (2008) Dbf4-dependent CDC7 kinase links DNA replication to the segregation of homologous chromosomes in meiosis I. Cell 135, 662–678 10.1016/j.cell.2008.10.026 19013276

[59] Lam, I. and Keeney, S. (2014) Mechanism and regulation of meiotic recombination initiation. Cold Spring Harb. Perspect. Biol. 7, a016634 10.1101/cshperspect.a016634 25324213 PMC4292169

[60] Claeys Bouuaert, C., Pu, S., Wang, J., Oger, C., Daccache, D., Xie, W. et al. (2021) DNA-driven condensation assembles the meiotic DNA break machinery. Nature 592, 144–149 10.1038/s41586-021-03374-w 33731927 PMC8016751

[61] Boekhout, M., Karasu, M.E., Wang, J., Acquaviva, L., Pratto, F., Brick, K. et al. (2019) REC114 partner ANKRD31 controls number, timing, and location of meiotic DNA breaks. Mol. Cell 74, 1053–68.e8 10.1016/j.molcel.2019.03.023 31003867 PMC6555648

[62] Papanikos, F., Clément, J.A.J., Testa, E., Ravindranathan, R., Grey, C., Dereli, I. et al. (2019) Mouse ANKRD31 regulates spatiotemporal patterning of meiotic recombination initiation and ensures recombination between X and Y sex chromosomes. Mol. Cell 74, 1069–1085.e11 10.1016/j.molcel.2019.03.022 31000436

[63] Laroussi, H., Juarez-Martinez, A.B., Le Roy, A., Boeri Erba, E., Gabel, F., de Massy, B. et al. (2023) Characterization of the REC114-MEI4-IHO1 complex regulating meiotic DNA double-strand break formation. EMBO J. 42, e113866 10.15252/embj.2023113866 37431931 PMC10425845

[64] Nore, A., Juarez-Martinez, A.B., Clément, J., Brun, C., Diagouraga, B., Laroussi, H. et al. (2022) TOPOVIBL-REC114 interaction regulates meiotic DNA double-strand breaks. Nat. Commun. 13, 1–19. 10.1038/s41467-022-34799-0 36396648 PMC9671922

[65] Alani, E., Padmore, R. and Kleckner, N. (1990) Analysis of wild-type and rad50 mutants of yeast suggests an intimate relationship between meiotic chromosome synapsis and recombination. Cell 61, 419–436 10.1016/0092-8674(90)90524-i 2185891

[66] Nairz, K. and Klein, F. (1997) Mre11s–a yeast mutation that blocks double-strand-break processing and permits nonhomologous synapsis in meiosis. Genes Dev. 11, 2272–2290 10.1101/gad.11.17.2272 9303542 PMC275393

[67] Chin, G.M. and Villeneuve, A.M. (2001) *C. elegans* mre-11 is required for meiotic recombination and DNA repair but is dispensable for the meiotic G(2) DNA damage checkpoint. Genes Dev. 15, 522–534 10.1101/gad.864101 11238374 PMC312651

[68] Puizina, J., Siroky, J., Mokros, P., Schweizer, D. and Riha, K. (2004) Mre11 deficiency in Arabidopsis is associated with chromosomal instability in somatic cells and Spo11-dependent genome fragmentation during meiosis. Plant Cell 16, 1968–1978 10.1105/tpc.104.022749 15258261 PMC519189

[69] Young, J.A., Hyppa, R.W. and Smith, G.R. (2004) Conserved and nonconserved proteins for meiotic DNA breakage and repair in yeasts. Genetics 167, 593–605 10.1534/genetics.103.023762 15238514 PMC1470912

[70] Vrielynck, N., Schneider, K., Rodriguez, M., Sims, J., Chambon, A., Hurel, A. et al. (2021) Conservation and divergence of meiotic DNA double strand break forming mechanisms in *Arabidopsis thaliana*. Nucleic Acids Res. 49, 9821–9835 10.1093/nar/gkab715 34458909 PMC8464057

[71] Henderson, K.A., Kee, K., Maleki, S., Santini, P.A. and Keeney, S. (2006) Cyclin-dependent kinase directly regulates initiation of meiotic recombination. Cell 125, 1321–1332 10.1016/j.cell.2006.04.039 16814718 PMC1950680

[72] Bhagwat, N.R., Owens, S.N., Ito, M., Boinapalli, J.V., Poa, P., Ditzel, A. et al. (2021) SUMO is a pervasive regulator of meiosis. Elife 10, e57720 10.7554/eLife.57720 33502312 PMC7924959

[73] Elbakry, A. and Löbrich, M. (2021) Homologous recombination subpathways: a tangle to resolve. Front. Genet. 12, 723847 10.3389/fgene.2021.723847 34408777 PMC8365153

[74] Gnügge, R. and Symington, L.S. (2021) DNA end resection during homologous recombination. Curr. Opin. Genet. Dev. 71, 99–105 10.1016/j.gde.2021.07.004 34329854 PMC9006674

[75] Kawale, A.S. and Sung, P. (2020) Mechanism and significance of chromosome damage repair by homologous recombination. Essays Biochem. 64, 779–790 10.1042/EBC20190093 32756864

[76] Ranjha, L., Howard, S.M. and Cejka, P. (2018 Jun) Main steps in DNA double-strand break repair: an introduction to homologous recombination and related processes. Chromosoma 127, 187–214 10.1007/s00412-017-0658-1 29327130

[77] Hayase, A., Takagi, M., Miyazaki, T., Oshiumi, H., Shinohara, M. and Shinohara, A. (2004) A protein complex containing Mei5 and Sae3 promotes the assembly of the meiosis-specific RecA Homolog Dmc1. Cell 119, 927–940. 10.1016/j.cell.2004.10.031 15620352

[78] Hinch, A.G., Becker, P.W., Li, T., Moralli, D., Zhang, G., Bycroft, C. et al. (2020) The configuration of RPA, RAD51, and DMC1 binding in meiosis reveals the nature of critical recombination intermediates. Mol. Cell 79, 689–701.e10 10.1016/j.molcel.2020.06.015 32610038 PMC7447979

[79] Chen, Y.K., Leng, C.H., Olivares, H., Lee, M.H., Chang, Y.C., Kung, W.M. et al. (2004) Heterodimeric complexes of Hop2 and Mnd1 function with Dmc1 to promote meiotic homolog juxtaposition and strand assimilation. Proc. Natl Acad. Sci. U.S.A. 101, 10572–10577 10.1073/pnas.0404195101 15249670 PMC490024

[80] Petukhova, G.V., Pezza, R.J., Vanevski, F., Ploquin, M., Masson, J.Y. and Camerini-Otero, R.D. (2005) The Hop2 and Mnd1 proteins act in concert with Rad51 and Dmc1 in meiotic recombination. Nat. Struct. Mol. Biol. 12, 449–453 10.1038/nsmb923 15834424

[81] Souquet, B., Abby, E., Hervé, R., Finsterbusch, F., Tourpin, S., Le Bouffant, R. et al. (2013) MEIOB targets single-strand DNA and is necessary for meiotic recombination. PLoS Genet. 9, e1003784. 10.1371/journal.pgen.1003784 24068956 PMC3778009

[82] Luo, M., Yang, F., Leu, N.A., Landaiche, J., Handel, M.A., Benavente, R. et al. (2013) MEIOB exhibits single-stranded DNA-binding and exonuclease activities and is essential for meiotic recombination. Nat. Commun. 4, 2788 10.1038/ncomms3788 24240703 PMC3891831

[83] Ribeiro, J., Dupaigne, P., Petrillo, C., Ducrot, C., Duquenne, C., Veaute, X. et al. (2021 Jun) The meiosis-specific MEIOB-SPATA22 complex cooperates with RPA to form a compacted mixed MEIOB/SPATA22/RPA/ssDNA complex. DNA Repair 102, 103097 10.1016/j.dnarep.2021.103097 33812231

[84] Zhang, J., Nandakumar, J. and Shibuya, H. (2022) BRCA2 in mammalian meiosis. Trends Cell Biol. 32, 281–284. 10.1016/j.tcb.2021.09.003 34625364

[85] Brandsma, I., Sato, K., van Rossum-Fikkert, S.E., van Vliet, N., Sleddens, E., Reuter, M. et al. (2019) HSF2BP interacts with a conserved domain of BRCA2 and is required for mouse spermatogenesis. Cell Rep. 27, 3790–3798.e7 10.1016/j.celrep.2019.05.096 31242413

[86] Zhang, J., Fujiwara, Y., Yamamoto, S. and Shibuya, H. (2019) A meiosis-specific BRCA2 binding protein recruits recombinases to DNA double-strand breaks to ensure homologous recombination. Nat. Commun. 10, 722 10.1038/s41467-019-08676-2 30760716 PMC6374363

[87] Felipe-Medina, N., Caburet, S., Sánchez-Sáez, F., Condezo, Y.B., de Rooij, D.G., Gómez-H, L. et al. (2020) A missense in HSF2BP causing primary ovarian insufficiency affects meiotic recombination by its novel interactor C19ORF57/BRME1. Elife 9, e56996 10.7554/eLife.56996 32845237 PMC7498267

[88] Takemoto, K., Tani, N., Takada-Horisawa, Y., Fujimura, S., Tanno, N., Yamane, M. et al. (2020) Meiosis-specific C19orf57/4930432K21Rik/BRME1 modulates localization of RAD51 and DMC1 to DSBs in mouse meiotic recombination. Cell Rep. 31, 107686 10.1016/j.celrep.2020.107686 32460033

[89] Zhang, J., Gurusaran, M., Fujiwara, Y., Zhang, K., Echbarthi, M., Vorontsov, E. et al. (2020) The BRCA2-MEILB2-BRME1 complex governs meiotic recombination and impairs the mitotic BRCA2-RAD51 function in cancer cells. Nat. Commun. 11, 1–18. 10.1038/s41467-019-13993-7 32345962 PMC7188823

[90] Shang, Y., Huang, T., Liu, H., Liu, Y., Liang, H., Yu, X. et al. (2020) MEIOK21: a new component of meiotic recombination bridges required for spermatogenesis. Nucleic Acids Res. 48, 6624–6639 10.1093/nar/gkaa406 32463460 PMC7337969

[91] Pendlebury, D.F., Zhang, J., Agrawal, R., Shibuya, H. and Nandakumar, J. (2021 Aug) Structure of a meiosis-specific complex central to BRCA2 localization at recombination sites. Nat. Struct. Mol. Biol. 28, 671–680 10.1038/s41594-021-00635-0 34373645 PMC8462358

[92] Bzymek, M., Thayer, N.H., Oh, S.D., Kleckner, N. and Hunter, N. (2010) Double Holliday junctions are intermediates of DNA break repair. Nature 464, 937–941. 10.1038/nature08868 20348905 PMC2851831

[93] Kim, K.P., Weiner, B.M., Zhang, L., Jordan, A., Dekker, J. and Kleckner, N. (2010) Sister cohesion and structural axis components mediate homolog bias of meiotic recombination. Cell 143, 924–937 10.1016/j.cell.2010.11.015 21145459 PMC3033573

[94] Lao, J.P. and Hunter, N. (2010) Trying to avoid your sister. PLoS Biol. 8, e1000519 10.1371/journal.pbio.1000519 20976046 PMC2957405

[95] Hong, S., Sung, Y., Yu, M., Lee, M., Kleckner, N. and Kim, K.P. (2013) The logic and mechanism of homologous recombination partner choice. Mol. Cell 51, 440–453 10.1016/j.molcel.2013.08.008 23973374 PMC4049084

[96] Xie, C., He, C., Jiang, Y., Yu, H., Cheng, L., Nshogoza, G. et al. (2018) Structural insights into the recognition of phosphorylated Hop1 by Mek1. Acta Cryst. 74, 1027–1038. 10.1107/S2059798318011993 30289413

[97] Carballo, J.A., Johnson, A.L., Sedgwick, S.G. and Cha, R.S. (2008) Phosphorylation of the axial element protein Hop1 by Mec1/Tel1 ensures meiotic interhomolog recombination. Cell 132, 758–770. 10.1016/j.cell.2008.01.035 18329363

[98] Niu, H., Wan, L., Baumgartner, B., Schaefer, D., Loidl, J. and Hollingsworth, N.M. (2005) Partner choice during meiosis is regulated by Hop1-promoted dimerization of Mek1. Mol. Biol. Cell 16, 5804–5818 10.1091/mbc.E05 16221890 PMC1289423

[99] Niu, H., Li, X., Job, E., Park, C., Moazed, D., Gygi, S.P. et al. (2007) Mek1 kinase is regulated to suppress double-strand break repair between sister chromatids during budding yeast meiosis. Mol. Cell. Biol. 27, 5456–5467 10.1128/MCB.00416-07 17526735 PMC1952091

[100] Niu, H., Wan, L., Busygina, V., Kwon, Y., Allen, J.A., Li, X. et al. (2009) Regulation of meiotic recombination via Mek1-mediated Rad54 phosphorylation. Mol Cell 36, 393–404 10.1016/j.molcel.2009.09.029 19917248 PMC2788773

[101] Callender, T.L., Laureau, R., Wan, L., Chen, X., Sandhu, R., Laljee, S. et al. (2016) Mek1 down regulates Rad51 activity during yeast meiosis by phosphorylation of Hed1. PLoS Genet. 12, e1006226 10.1371/journal.pgen.1006226 27483004 PMC4970670

[102] Goldfarb, T. and Lichten, M. (2010) Frequent and efficient use of the sister chromatid for DNA double-strand break repair during budding yeast meiosis. PLoS Biol. 8, e1000520 10.1371/journal.pbio.1000520 20976044 PMC2957403

[103] Chen, X., Gaglione, R., Leong, T., Bednor, L., de Los Santos, T., Luk, E. et al. (2018) Mek1 coordinates meiotic progression with DNA break repair by directly phosphorylating and inhibiting the yeast pachytene exit regulator Ndt80. PLoS Genet. 14, e1007832 10.1371/journal.pgen.1007832 30496175 PMC6289461

[104] Hollingsworth, N.M. and Gaglione, R. (2019) The meiotic-specific Mek1 kinase in budding yeast regulates interhomolog recombination and coordinates meiotic progression with double-strand break repair. Curr. Genet. 65, 631–641 10.1007/s00294-019-00937-3 30671596 PMC6511291

[105] Lisby, M. and Rothstein, R. (2015) Cell biology of mitotic recombination. Cold Spring Harb. Perspect. Biol. 7, a016535 10.1101/cshperspect.a016535 25731763 PMC4355273

[106] West, A.M.V., Komives, E.A. and Corbett, K.D. (2017) Conformational dynamics of the Hop1 HORMA domain reveal a common mechanism with the spindle checkpoint protein Mad2. Nucleic Acids Res. 46, 279–292 10.1093/nar/gkx1196 PMC5758881 29186573

[107] Aravind, L. and Koonin, E.V. (1998) The HORMA domain: a common structural denominator in mitotic checkpoints, chromosome synapsis and DNA repair. Trends Biochem. Sci. 23, 284–286 10.1016/s0968-0004(98)01257-2 9757827

[108] Daccache, D., De Jonge, E., Liloku, P., Mechleb, K., Haddad, M. and Corthaut, S. et al. (2023) Evolutionary conservation of the structure and function of meiotic Rec114−Mei4 and Mer2 complexes. Genes Dev. 37, 535–553 10.1101/gad.350462.123 37442581 PMC10393190

[109] Kumar, R., Oliver, C., Brun, C., Juarez-Martinez, A.B., Tarabay, Y., Kadlec, J. et al. (2018) Mouse REC114 is essential for meiotic DNA double-strand break formation and forms a complex with MEI4. Life Sci. Alliance 1, e201800259 10.26508/lsa.201800259 30569039 PMC6288613

[110] Liu, K., Grasso, E.M., Pu, S., Zou, M., Liu, S., Eliezer, D. et al. (2023) Structure and DNA-bridging activity of the essential Rec114-Mei4 trimer interface. Genes Dev. 37, 518–534 10.1101/gad.350461.123 37442580 PMC10393192

[111] Carballo, J.A., Panizza, S., Serrentino, M.E., Johnson, A.L., Geymonat, M., Borde, V. et al. (2013) Budding yeast ATM/ATR control meiotic double-strand break (DSB) levels by down-regulating Rec114, an essential component of the DSB-machinery. PLoS Genet. 9, e1003545 10.1371/journal.pgen.1003545 23825959 PMC3694840

[112] Keeney, S., Giroux, C.N. and Kleckner, N. (1997) Meiosis-specific DNA double-strand breaks are catalyzed by Spo11, a member of a widely conserved protein family. Cell 88, 375–384 10.1016/s0092-8674(00)81876-0 9039264

[113] Bergerat, A., de Massy, B., Gadelle, D., Varoutas, P.C., Nicolas, A. and Forterre, P. (1997) An atypical topoisomerase II from archaea with implications for meiotic recombination. Nature 386, 414–417. 10.1038/386414a0 9121560

[114] Mazina, O.M., Mazin, A.V., Nakagawa, T., Kolodner, R.D. and Kowalczykowski, S.C. (2004) *Saccharomyces cerevisiae* Mer3 Helicase Stimulates 3′–5′ Heteroduplex Extension by Rad51. Cell. 117, 47–56 10.1016/s0092-8674(04)00294-6 15066281

[115] Adams, I.R. and Davies, O.R. (2023) Meiotic chromosome structure, the synaptonemal complex, and infertility. Annu. Rev. Genom. Hum. Genet. 24, 35–61 10.1146/annurev-genom-110122-090239 37159901

[116] Arora, K. and Corbett, K.D. (2018) The conserved XPF:ERCC1-like Zip2:Spo16 complex controls meiotic crossover formation through structure-specific DNA binding. Nucleic Acids Res. 47, 2365–2376 10.1093/nar/gky1273 PMC6411835 30566683

[117] Durand, S., Lian, Q., Jing, J., Ernst, M., Grelon, M., Zwicker, D. et al. (2022) Joint control of meiotic crossover patterning by the synaptonemal complex and HEI10 dosage. Nat. Commun. 13, 5999 10.1038/s41467-022-33472-w 36224180 PMC9556546

[118] Lamers, M.H., Perrakis, A., Enzlin, J.H., Winterwerp, H.H., de Wind, N. and Sixma, T.K. (2000) The crystal structure of DNA mismatch repair protein MutS binding to a G x T mismatch. Nature. 407, 711–717 10.1038/35037523 11048711

[119] Lahiri, S., Li, Y., Hingorani, M.M. and Mukerji, I. (2018) MutSγ-induced DNA conformational changes provide insights into its role in meiotic recombination. Biophys. J. 115, 2087–2101 10.1016/j.bpj.2018.10.029 30467025 PMC6289823

[120] Snowden, T., Acharya, S., Butz, C., Berardini, M. and Fishel, R. (2004) hMSH4-hMSH5 recognizes Holliday Junctions and forms a meiosis-specific sliding clamp that embraces homologous chromosomes. Mol. Cell 15, 437–451 10.1016/j.molcel.2004.06.040 15304223

[121] Börner, G.V., Kleckner, N. and Hunter, N. (2004) Crossover/noncrossover differentiation, synaptonemal complex formation, and regulatory surveillance at the leptotene/zygotene transition of meiosis. Cell 117, 29–45 10.1016/s0092-8674(04)00292-2 15066280

[122] Pyatnitskaya, A., Borde, V. and Muyt, A.D. (2019) Crossing and zipping: molecular duties of the ZMM proteins in meiosis. Chromosoma 128, 181–198 10.1007/s00412-019-00714-8 31236671

[123] Allan, R.K. and Ratajczak, T. (2011) Versatile TPR domains accommodate different modes of target protein recognition and function. Cell Stress Chaperones 16, 353–367 10.1007/s12192-010-0248-0 21153002 PMC3118826

[124] Pyatnitskaya, A., Andreani, J., Guérois, R., De Muyt, A. and Borde, V. (2022) The Zip4 protein directly couples meiotic crossover formation to synaptonemal complex assembly. Genes Dev. 36, 53–69 10.1101/gad.348973.121 34969823 PMC8763056

[125] Pena, V., Jovin, S.M., Fabrizio, P., Orlowski, J., Bujnicki, J.M., Lührmann, R. et al. (2009) Common design principles in the spliceosomal RNA helicase Brr2 and in the Hel308 DNA helicase. Mol. Cell 35, 454–466 10.1016/j.molcel.2009.08.006 19716790

[126] Duroc, Y., Kumar, R., Ranjha, L., Adam, C., Guérois, R., Md Muntaz, K. et al. (2017) Concerted action of the MutLβ heterodimer and Mer3 helicase regulates the global extent of meiotic gene conversion. Elife 6, e21900 10.7554/eLife.21900 28051769 PMC5215242

[127] Storlazzi, A., Gargano, S., Ruprich-Robert, G., Falque, M., David, M., Kleckner, N. et al. (2010) Recombination proteins mediate meiotic spatial chromosome organization and pairing. Cell 141, 94–106 10.1016/j.cell.2010.02.041 20371348 PMC2851631

[128] Rao, H.B.D.P., Qiao, H., Bhatt, S.K., Bailey, L.R.J., Tran, H.D., Bourne, S.L. et al. (2017) A SUMO-ubiquitin relay recruits proteasomes to chromosome axes to regulate meiotic recombination. Science 355, 403–407 10.1126/science.aaf6407 28059716 PMC5569317

[129] Nakagawa, T. and Kolodner, R.D. (2002) The MER3 DNA helicase catalyzes the unwinding of holliday junctions. J. Biol. Chem. 277, 28019–28024 10.1074/jbc.M204165200 12039965

[130] Vernekar, D.V., Reginato, G., Adam, C., Ranjha, L., Dingli, F., Marsolier, M.C. et al. (2021) The Pif1 helicase is actively inhibited during meiotic recombination which restrains gene conversion tract length. Nucleic Acids Res. 49, 4522–4533 10.1093/nar/gkab232 33823531 PMC8096244

[131] Woglar, A. and Villeneuve, A.M. (2018) Dynamic architecture of DNA repair complexes and the synaptonemal complex at sites of meiotic recombination. Cell 173, 1678–91.e16 10.1016/j.cell.2018.03.066 29754818 PMC6003859

[132] Zakharyevich, K., Tang, S., Ma, Y. and Hunter, N. (2012) Delineation of joint molecule resolution pathways in meiosis identifies a crossover-specific resolvase. Cell 149, 334–347. 10.1016/j.cell.2012.03.023 22500800 PMC3377385

[133] Manhart, C.M., Ni, X., White, M.A., Ortega, J., Surtees, J.A. and Alani, E. (2017 Apr) The mismatch repair and meiotic recombination endonuclease Mlh1-Mlh3 is activated by polymer formation and can cleave DNA substrates in trans. PLoS Biol. 15, e2001164 10.1371/journal.pbio.2001164 28453523 PMC5409509

[134] Ranjha, L., Anand, R. and Cejka, P. (2014) The *Saccharomyces cerevisiae* Mlh1-Mlh3 heterodimer is an endonuclease that preferentially binds to holliday junctions. J. Biol. Chem. 289, 5674–5686. 10.1074/jbc.M113.533810 24443562 PMC3937642

[135] Cannavo, E., Sanchez, A., Anand, R., Ranjha, L., Hugener, J., Adam, C. et al. (2020) Regulation of the MLH1-MLH3 endonuclease in meiosis. Nature 586, 618–622 10.1038/s41586-020-2592-2 32814904

[136] Kulkarni, D.S., Owens, S.N., Honda, M., Ito, M., Yang, Y., Corrigan, M.W. et al. (2020) PCNA activates the MutLγ endonuclease to promote meiotic crossing over. Nature. 586, 623–627 10.1038/s41586-020-2645-6 32814343 PMC8284803

[137] Dubois, E., Muyt, A.D., Soyer, J.L., Budin, K., Legras, M., Piolot, T. et al. (2019) Building bridges to move recombination complexes. Proc. Natl Acad. Sci. U.S.A. 116, 12400–12409 10.1073/pnas.1901237116 31147459 PMC6589682

[138] Dunce, J.M., Salmon, L.J. and Davies, O.R. (2021) Structural basis of meiotic chromosome synaptic elongation through hierarchical fibrous assembly of SYCE2-TEX12. Nat. Struct. Mol. Biol. 28, 681–693 10.1038/s41594-021-00636-z 34373646 PMC7612376

[139] Girard, C., Zwicker, D. and Mercier, R. (2023) The regulation of meiotic crossover distribution: a coarse solution to a century-old mystery? Biochem. Soc. Trans. 51, 1179–1190 10.1042/BST20221329 37145037 PMC10317170

[140] Chelysheva, L., Vezon, D., Chambon, A., Gendrot, G., Pereira, L., Lemhemdi, A. et al. (2012) The Arabidopsis HEI10 is a new ZMM protein related to Zip3. PLoS Genet. 8, e1002799 10.1371/journal.pgen.1002799 22844245 PMC3405992

[141] Toby, G.G., Gherraby, W., Coleman, T.R. and Golemis, E.A. (2003) A novel RING finger protein, human enhancer of invasion 10, alters mitotic progression through regulation of cyclin B levels. Mol. Cell. Biol. 23, 2109–2122 10.1128/MCB.23.6.2109-2122.2003 12612082 PMC149478

[142] Reynolds, A., Qiao, H., Yang, Y., Chen, J.K., Jackson, N., Biswas, K. et al. (2013) RNF212 is a dosage-sensitive regulator of crossing-over during mammalian meiosis. Nat. Genet. 45, 269–278 10.1038/ng.2541 23396135 PMC4245152

[143] Morgan, C., Fozard, J.A., Hartley, M., Henderson, I.R., Bomblies, K. and Howard, M. (2021) Diffusion-mediated HEI10 coarsening can explain meiotic crossover positioning in Arabidopsis. Nat. Commun. 12, 1–11. 10.1038/s41467-020-20314-w 34344879 PMC8333306

[144] Zhang, L., Stauffer, W., Zwicker, D. and Dernburg, A. F. (2021) Crossover patterning through kinase-regulated condensation and coarsening of recombination nodules. bioRxiv 2021.08.26.457865. https://www.biorxiv.org/content/10.1101/2021.08.26.457865v1

[145] Jumper, J., Evans, R., Pritzel, A., Green, T., Figurnov, M., Ronneberger, O. et al. (2021) Highly accurate protein structure prediction with AlphaFold. Nature 596, 583–589 10.1038/s41586-021-03819-2 34265844 PMC8371605

